# Stranger danger or good Samaritan? A cross-sectional study examining correlates of tolerance of risk in outdoor play among Canadian parents

**DOI:** 10.1186/s12889-025-21848-8

**Published:** 2025-02-14

**Authors:** Guy Faulkner, Matthew Fagan, Julia McKenna, Mariana Brussoni, Mathieu Bélanger, Katie Gunnell, Mark S. Tremblay, Richard Larouche

**Affiliations:** 1 School of Kinesiology, University of British Columbia, Vancouver, Canada; 2 Department of Pediatrics, School of Population and Public Health, Human Early Learning Partnership, University of British Columbia, British Columbia Children’s Hospital Research Institute, Vancouver, Canada; 3 Faculté de médecine et des sciences de la santé, Université de Sherbrooke, Sherbrooke, Canada; 4 Department of Psychology, Carleton University, Ottawa, Canada; 5 Healthy Active Living and Obesity Research Group, CHEO Research Institute, Ottawa, Canada; 6 Faculty of Health Sciences, University of Lethbridge, Lethbridge, Canada

**Keywords:** Risk, Parents, Outdoor play, Physical activity

## Abstract

**Background:**

Negative parental perceptions of risk may restrict children’s opportunities for outdoor play. Excessively minimizing children’s exposure to risks in their environment may have a range of developmental consequences. The purpose of this cross-sectional study was to assess correlates of parental tolerance of risk among a large sample of Canadian parents.

**Methods:**

In this cross-sectional study, a sample of 2,291 parents of 7–12 year olds completed online questionnaires assessing a range of potential individual (e.g., gender), social (e.g., neighbourhood cohesion), and environmental (e.g., walkability) correlates of parental tolerance of risk. Logistic regressions were created to examine associations between these factors and odds of being in the most risk averse quartile. The logistic regression was built in hierarchal steps relying on the Akaike information criterion (AIC) and pseudo R^2^ for model progression.

**Results:**

The final model had a pseudo R^2^ of 0.18. Five out of seventeen correlates were associated with risk aversion in parents. Concerns about stranger danger were associated with a higher odds of risk aversion (OR = 2.33, 95%CI[1.93, 2.82]). A higher number of children in the home was associated with lower odds of risk aversion in parents (OR = 0.80, 95%CI[0.69, 0.92], and parents of children born outside of Canada had higher odds of being risk adverse when compared to parents born in Canada (OR = 2.13, 95%CI[1.54, 2.94]). Finally, being very concerned with COVID-19 increased the odds of risk aversion (OR = 3.07, 95%CI[1.93, 5.04], while having a household income of > 100,000 lowered the odds of risk aversion (OR = 0.56, 95%CI[0.36, 0.87]).

**Conclusions:**

Tailored interventions that reframe perceptions of risk for parents are needed. Such interventions could reframe concerns about stranger danger which persist despite occurrences of stranger abduction being extremely rare. Interventions could also be targeted to immigrant families and those with fewer children as they appear to be more averse to risk. A complementary focus on examining how cultural background influences risk perceptions is needed in future research.

**Supplementary Information:**

The online version contains supplementary material available at 10.1186/s12889-025-21848-8.

## Introduction

Physical activity (PA) provides numerous social, physical, and mental health benefits for children and youth. PA is associated with improved cardiorespiratory fitness, bone health, cardiometabolic health, and cognitive function, as well as a reduced risk of experiencing depression [1, 2]. Despite the benefits, the 2022 Canadian Report Card on Physical Activity for Children and Youth indicated only 17.5% of children ages 5–11 years were meeting the *Canadian 24-Hour Movement Guidelines for Children and Youth* recommendation to accumulate 60 min of daily moderate-to-vigorous intensity PA [3]. Across 57 countries, ‘Overall Physical Activity’ was the indicator with the lowest average grade (D) corresponding to an estimation of only 27–33% of children and adolescents meeting the recommended amount of moderate-to-vigorous intensity PA [4].

Some sources of PA include outdoor active play (i.e., a form of play that involves PA of any intensity that occurs outdoors; 5) and active transportation (i.e., walking, biking, or running to/from a location). According to the aforementioned report card, just 33% of 5- to 11-year-old Canadian children were engaging in > 2 h/day in outdoor active play [3]. Outdoor active play and active transportation are influenced by independent mobility (IM; a child’s freedom to move around in public space without adult supervision; 6). For example, a Canadian, multi-site cross-sectional study found that IM was associated with increased levels of active travel and PA across all sites [7]; however, there has been a stark decline in children’s IM and active transport to and from school over the past several decades [6]. One study found that the percentage of primary school children in England who were allowed to travel to school alone decreased from 86% in 1971 to just 25% in 2010 [8]. In another study of neighbourhood activity among Canadian children aged 9–13 years, 94% of their free time was spent less than 400 m away from their homes, implying that children may be increasingly restricted in their ability to be active outside of the house [9].

The low and declining participation in active play [4, 10] and restriction in IM could be related to heightened parental perceptions of risk. Risk involves engaging with uncertainty– exposing oneself to an activity or other stimuli with a possible outcome that can be either positive or negative [11]. Risk can be applied to many different settings where children find themselves, such as at a playground, travelling to school independently, and going to the park. In the context of play, risky play involves experimenting with uncertainty and overcoming fears [12]. Examples include playing at height (e.g., climbing a tree), playing with dangerous tools, playing near dangerous elements (e.g., water, fire), rough-and-tumble play (e.g., play fighting), and play where children have the potential to get lost (including IM) [13]. Adult anxieties about harm to the child, legal liability, and blame may make it difficult for parents to strike the right balance between children having the freedom to play the way they choose, including risky play, and keeping them safe from harm. As such, decreases in outdoor active play and IM may be, in part, due to the emergence of parents “bubble-wrapping” their children, in which a lower parental tolerance of risk (PToR) results in excessively minimizing children’s exposure to risks in their environment [14]. Malone [14] suggested that parents’ perceptions of risk have heightened over time due to the media sensationalization of ‘stranger danger’, the globalization of terrorism, and fear of being seen as a neglectful parent. Others have pointed to broader societal shifts toward ‘intensive parenting’ where parents (primarily mothers) are encouraged to become experts on parenting strategies that may minimize exposure to risk [15]. Of course, such shifts are a consequence of complex social and economic processes that have resulted from changing population age distributions, the nature of work (e.g., multi-worker households), the composition of families (e.g., smaller families), and mobility itself (e.g., increasing auto-ownership and suburbanization) [16].

Despite good intentions, instead of protecting children from safety concerns during outdoor active play and IM, such as physical injury, ‘stranger danger,’ and the possibility of getting lost [6, 17], low PToR may be harming children by reducing developmentally necessary opportunities to learn to identify and manage risks [18], and engage in outdoor risky play [19]. One way to measure risk is with the Tolerance for Risk in Play Scale (TRiPS), a 31-item survey that assesses one’s risk tolerance towards activities during children’s play [20]. Items for the TRiPS largely reflect the six categories of risky play described by Sandseter [12, 13]. The items range from those most people would deem ‘very risky’ (e.g., Would you let the child play near the edge of steep cliffs? ) to items many would deem ‘not risky at all’ (Would you let your child play tag with other children? ). In their initial validation work [20], the scale scores demonstrated evidence for internal and external validity, and internal reliability. Importantly, responses to the scale differentiated among adults who were more and less tolerant to risk in children’s play.

Using this scale, two large national surveys have examined parents’ tolerance of risk and its relationship to associated constructs such as IM and outdoor play. In a nationally representative sample of 2,003 New Zealand parents, Jelleyman et al. [21] assessed the attitudes of parents towards risky play practices and IM, the barriers preventing them from allowing their children to participate, and how often their children engaged in risky play activities. They found that while parents believed that risky play is beneficial for children’s development they also did not allow their children to participate in such activities. These authors call for more research identifying which socio-demographic and individual characteristics influence risky play practices. In another study, the British Children’s Play Survey [19] of 1,919 parents/caregivers with a child aged 5–11 years assessed the socio-demographic factors, geographic factors and parental attitudes to risk and protection associated with their children’s total time spent playing and engaging in IM. Parents who reported they were more tolerant of risk also reported that their children spent more total time playing, more time playing outdoors, and more time playing adventurously. Parents with higher risk tolerance scores also had children who had greater IM at a given age. This research highlights the role of risk tolerance as an important correlate of outdoor play and IM, but there was no specific examination of the socio-demographic and individual characteristics associated with risk tolerance itself.

Risk perception may differ in the culture, lived experiences, and practices of parents from different countries. For example, one study found that parents and educators from the USA and Australia tend to be more risk averse than those from Norway and Canada [22]. A more recent European survey reported that parents and early childhood educators from Norway were less aversive to risk in children’s play than those from Greece or Portugal [23]. The lack of population-based studies on parental tolerance to risk in Canada make it difficult to inform policies, interventions, and education campaigns to promote IM and outdoor active play. Such studies could also identify sub-groups that are more risk-averse and correlates of risk-aversion to help guide the development of effective interventions encouraging IM and outdoor active play.

One way to conceptualize correlates of tolerance of risk is through a social-ecological framework. A social-ecological framework demonstrates the various levels across which a health behaviour is influenced, and the dynamic interconnection of such influences [24]. Potential influences include factors at the individual (e.g., child’s age, child’s gender, child’s disability status, mobile phone ownership, years spent in Canada, parental age, parental gender, parental education level, socioeconomic status), social environment (e.g., number of children in the home, perceptions of traffic, neighbourhood, and crime safety), and built or physical environmental (e.g., region lived in, active-living friendliness or walkability score) levels. Accordingly, the objective of this study was to examine the individual, social, and built or physical environmental predictors of membership of the most risk-averse category of parents. We hypothesised that factors across the social-ecological framework most closely reflecting safety (e.g., perceptions of traffic, neighbourhood, and crime safety, walkability, child’s age) would be significant.

## Methods

### Participants and recruitment

We used baseline data collected in December 2020 from a national study assessing changes in movement behaviours and IM among children over time [25]. Data were collected by a market survey firm Léger (leger360.com). Léger collected baseline data in December 2020 from 2,291 parents of 7- to 12-year-olds through a self-administered online questionnaire (see supplementary file) using a computer-aided web interviewing method. The sample was designed to be demographically representative of Canadian parents based on education and household income. Only parents who: (1) could complete the survey in English or French; (2) had a 7- to 12-year-old child; and (3) agreed to be re-invited for 6-, 12-, and 18-month follow-ups were eligible. If parents had multiple children in the target age range, they were asked to answer the survey for the child whose name came first alphabetically. Parents received a CAD$3 compensation for each survey. The study protocol was approved by institutional research ethics committees, and parents consented electronically after reviewing an information letter.

### Measures

#### Outcome measure

The Tolerance of Risk in Play Scale (TRiPS) was used to assess parents’ tolerance of risk in the current work [20]. A 30-item version of the TRiPS questionnaire was used as item 21 (probing acceptability of play fighting with other children) was removed from the questionnaire (see 26). For each item, response options were “no” (0) and “yes” [1]. The 30-items were then summed to create an integer from 0 to 30 with higher scores representing a heightened risk tolerance.

#### Individual factors

Parents who completed the survey were asked to self-report their age (years), gender (man, woman, prefer not to answer, and other (specify)), highest education level (elementary or less, high school, college, university), and income ($39,999 or less, $40,000-$99,999, $100,000 or more). Parents also reported their child’s gender (boy, girl, “they identify as (specify)” and prefer not to say), age (years), disability status (yes vs. no), phone ownership (yes vs. no), COVID-19 concern (not concerned, moderately concerned, very concerned), and years lived in Canada (born in Canada, 2 years or less, 3 to 5 years, 6 years or more).

#### Social environment factors

Parents were asked questions about factors that would influence the social environment of their child. Such factors included the number of children living in the home, as well as their perceptions of neighbourhood social cohesion, traffic safety, crime safety, and stranger danger.

##### Social cohesion

We assessed neighbourhood social cohesion using a validated subscale from Sampson and colleagues’ [27] collective efficacy scale. Parents were asked to what extent they agreed or disagreed with five statements on a 4-point Likert scale (1 = *strongly disagree* and 4 = *strongly agree*). The statements were: (1) People around my neighbourhood are willing to help their neighbours; (2) This is a close-knit neighbourhood; (3) People in my neighbourhood can be trusted; (4) People in my neighbourhood generally don’t get along with each other; and (5) People in my neighbourhood do not share the same values, attitudes or beliefs. Questions 4 and 5 were reverse coded for easier interpretation. The Cronbach alpha was satisfactory = 0.77 (95%CI: 0.75, 0.79).

##### Perceived traffic safety

We employed subscales from the Neighbourhood Environment Walkability Scale– Youth [28] to assess parents’ concerns about traffic and crime in their neighbourhood. For traffic safety, parents were asked their agreement with three items on a 4-point Likert scale (1 = *strongly disagree* and 4 = *strongly agree*): (1) There is so much traffic along nearby streets that it makes it difficult or unpleasant for my child to walk (alone or with someone) in our neighbourhood; (2) The speed of traffic on most nearby streets is usually slow (50 km/h or less); and (3) Most drivers go faster than the posted speed limits in our neighbourhood. Question 2 was reverse coded, and items were averaged. The Cronbach alpha for traffic safety was 0.53 (95%CI: 0.49, 0.57).

##### Perceived crime safety

Crime safety (2-items) was created by calculating a value derived from summing the items divided by the number of items. Parents were asked their agreement on a 4-point Likert scale (1 = *strongly disagree* and 4 = *strongly agree*): (1) There is a high crime rate in our neighbourhood; and (2) The crime rate in our neighbourhood makes it unsafe for my child to go on walks (alone or with someone) at night. The Cronbach alpha for crime safety was 0.85 (95%CI: 0.83, 0.87).

##### Stanger danger

Stranger danger (4-items) was assessed with four items on a 4-point Likert scale (1 = *strongly disagree* and 4 = *strongly agree*): (1) I am worried about letting my child play outside *alone* around my home (e.g., yard, driveway, apartment common area) because I am afraid of them being taken or hurt by a stranger; (2) I am worried about letting my child be outside *with a friend* around my home because I am afraid my child will be taken or hurt by a stranger; (3) I am worried about letting my child play or walk alone or with friends in my neighbourhood and local streets because I am afraid my child will be taken or hurt by a stranger; and (4) I am worried about letting my child be alone or with friends in a local or nearby park because I am afraid my child will be taken or hurt by a stranger. The Cronbach alpha was 0.91 (95%CI: 0.90, 0.92).

##### Built environment factors

Parents reported their province or territory of residence and were asked to provide their 6-digit postal code. Using postal codes, we determined the walkability of their neighbourhood according to the Canadian Active Living Environments (Can-ALE) database [29]. Based on the z-scores of three variables (intersection density, dwelling density, and points of interest), Herrmann et al. [29] derived a 5-point variable (referred to as the Can-ALE class) ranking neighbourhood walkability from the least [1] to the most [5] walkable.

### Statistical analysis

#### Data cleaning procedures

All variables except the Can-ALE class had no missing data. Approximately 12% of the Can-ALE class scores could not be computed because participants did not report their postal code or misspelled it. Additionally, some individuals were excluded based on limited numbers of group membership. For example, our analysis only includes individuals who identified as men and women for parents; boys and girls for children. Our final analysis included a total of 1983 participants.

#### Rasch analysis

Our statistical analyses were conducted with R software. First, a Rasch analysis was completed for the outcome measure TRiPS with the *mrt* package. These scores were determined via a Rasch analysis as conducted within the original validation study for the TRiPS [20]. Theta standardized scores from the Rasch analysis ranged from − 0.9701 to 5.261 (SD = 1.823). A higher standardized score indicates higher tolerance of risky play. The Theta scores were highly correlated with the raw TRiPS scores (*r* = 0.98). Using the calculated Theta score for risk tolerances, quartiles were created to reflect four categories of risk tolerance [21]. Following the quartiles, a dichotomous variable of the most risk averse parents compared to the rest of the sample was computed.

#### Description of sample characteristics

The Wilcoxon Rank Sum Test for continuous variables and Fisher’s Exact Test for categorical variables were used to determine differences between the most risk averse and remaining parents [30, 31].

#### Logistic regression

Models were built to reflect individual factors (including age, gender, income, education, years lived in Canada, disability status, and phone ownership), social factors (including household composition, social cohesion, traffic safety, crime safety, and stranger danger), and environmental factors (including province of residence and walkability of neighborhood) employed within the social-ecological model. Using the *lme4* package, logistic regressions were created to assess associations between being highly risk averse and individual, social, and environmental factors. The logistic regression was built in hierarchal steps relying on the Akaike information criterion (AIC) and pseudo R^2^ for model progression. Additionally, the generalized VIF score was calculated to ensure absence of multicollinearity. Finally, after models were created, Benjamini Hochberg (BH) adjustments were made to the *p*-values through the *p.adjust(p*,* method=”BH”)* function in the *stats* package [32–33].

## Results

### Descriptive results

**Fig. 1:**
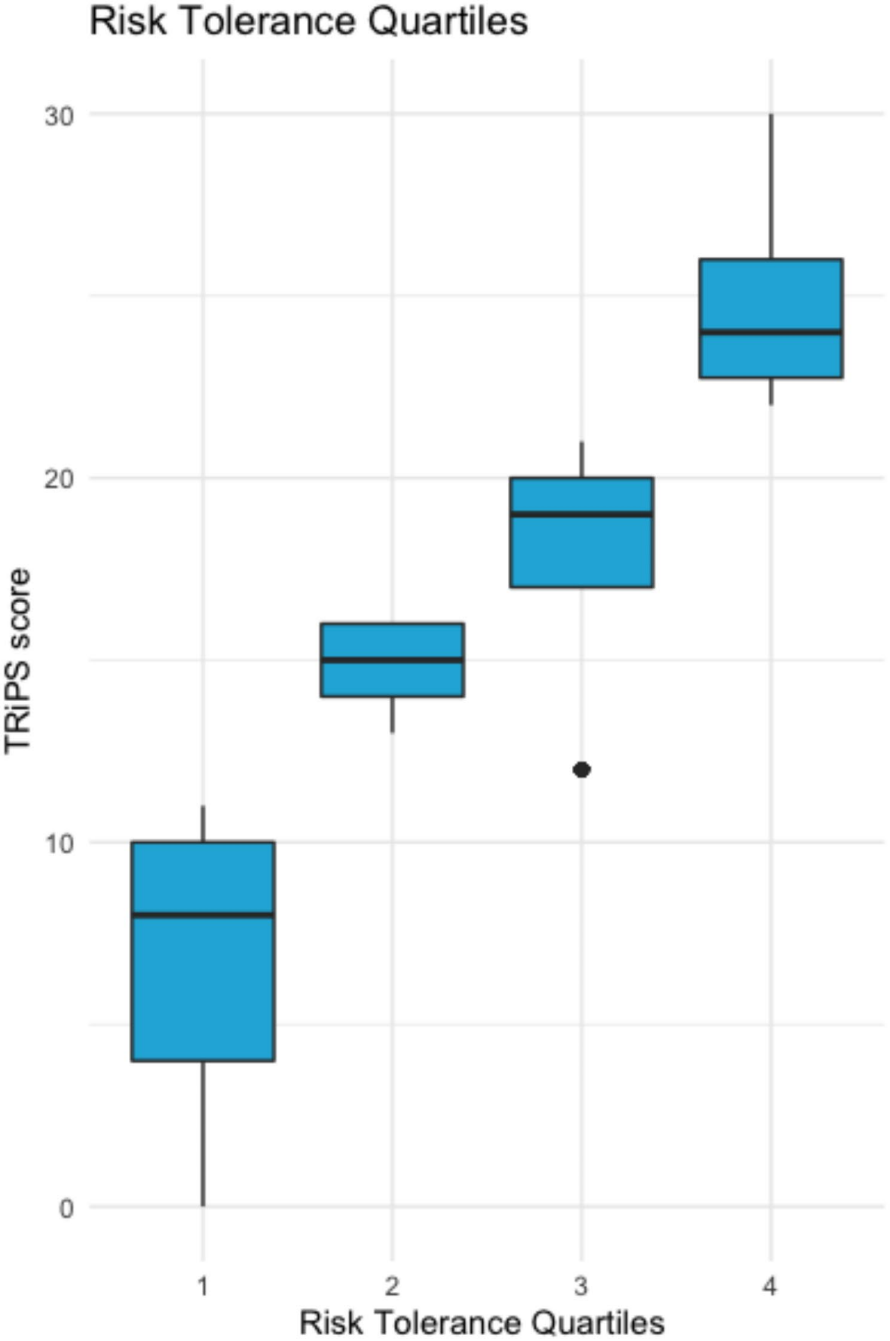
Scores on the TRiPS and quartiles of Theta scores from the Rasch analysis Note: TRiPS = The Tolerance for Risk in Play Scale

Figure 1 displays four quartiles of risk tolerance and the score on the TRiPS. Table 1 presents complete descriptive statistics for our sample comparing the most risk averse group of parents with the other risk tolerance categories. Demographic and descriptive statistics differed between parents who were most risk averse compared to the other parents. The average age of the child was younger and less likely to be born in Canada in the most risk averse group. Risk averse parents were younger, more likely to be a man, have a lower household income, had more COVID-19 concern, and report fewer children at home. These parents also reported lower social cohesion scores and higher concerns about traffic and crime safety, and stranger danger (all *p*’s < 0.05). Finally, risk averse parents lived in more walkable neighbourhoods based on the ALE (all *p*’s < 0.05).

**Table 1:** Descriptive statistics based on parental tolerance of risk

Characteristic	Not Risk Averse, *N* = 1,784^1^	Risk Averse, *N* = 490^1^	*p*-value^2^
**Child age**	9.95 (1.67)	9.66 (1.74)	0.001
**Child gender**			> 0.9
Boy	919 (52%)	253 (52%)	
Girl	865 (48%)	237 (48%)	
**Child Disability**			0.13
Yes	207 (12%)	45 (9.2%)	
No	1,577 (88%)	445 (91%)	
**Child phone ownership**			0.12
Yes	601 (34%)	147 (30%)	
No	1,183 (66%)	343 (70%)	
**Time in Canada**			< 0.001
Born in Canada	1,523 (85%)	346 (71%)	
2 years or less	196 (11%)	102 (21%)	
3 to 5 years	46 (2.6%)	26 (5.3%)	
6 years or more	19 (1.1%)	16 (3.3%)	
**Parent age**	41.37 (7.47)	40.51 (8.12)	0.031
**Parent gender**			0.024
Man	609 (34%)	198 (40%)	
Woman	1,172 (66%)	291 (59%)	
**COVID-19 concern**			< 0.001
Not concerned	280 (16%)	34 (7%)	
Somewhat concerned	1015 (57%)	217 (44%)	
Very Concerned	489 (27%)	239 (49%)	
**Parent education level**			
High school or less	290(16%)	93(19%)	
College	540(30%)	126(26%)	
University or more	908(51%)	258(53%)	
Prefer not to say	46(2.6%)	13(2.7%)	
**Household income**			< 0.001
<$39,999	184 (10%)	76 (16%)	
$40,000-$99,999	754 (42%)	268 (55%)	
>$100,000	674 (38%)	112 (23%)	
Prefer not to say	172 (9.6%)	34 (6.9%)	
**Number of children at home**	2.07(0.97)	1.82(0.86)	< 0.001
**Social cohesion (1–4)**	3.60 (0.73)	3.31 (0.69)	< 0.001
**Traffic safety (1–4)**	2.44 (0.65)	2.61 (0.57)	< 0.001
**Crime safety (1–4)**	2.09 (0.74)	2.66 (0.70)	< 0.001
**Stranger danger (1–4)**	2.22 (0.83)	2.87 (0.78)	< 0.001
**Region**			0.001
BC/Yukon	199 (11%)	63 (13%)	
Prairies	356 (20%)	83 (17%)	
Ontario	681 (38%)	203 (41%)	
Quebec	395 (22%)	123 (25%)	
Atlantic Canada	153 (8.6%)	18 (3.7%)	
**Walkability score (Can-ALE)**			< 0.001
1	498 (32%)	72 (17%)	
2	505 (32%)	129 (30%)	
3	370 (24%)	145 (34%)	
4	117 (7.5%)	59 (14%)	
5	67 (4.3%)	21 (4.9%)	

^1^ Mean (SD); n (%);^2^ Wilcoxon rank sum test; Fisher’s exact test

### Logistic regression model

Our models were built in three stages to reflect the social-ecological model (see Table 2 for a model fit including the AIC and McFadden pseudo R^2^ of the model stages). Our final model had a pseudo R^2^ of 0.1771. After controlling for multiple comparisons, five correlates were associated with risk aversion in parents (see Table 3). Concern about stranger danger was associated with higher odds of risk aversion (OR = 2.33, 95%CI[1.93, 2.82], BH adjusted *p*-value = < 0.01). More children in the home was inversely associated with odds of risk aversion in parents (OR = 0.80, 95%CI[0.69, 0.92], BH adjusted *p*-value = 0.009), and parents of a child born outside of Canada presented higher odds of being risk averse when compared to having one’s child born in Canada (OR = 2.13, 95%CI[1.54, 2.94], BH adjusted *p*-value = < 0.001). Finally, parents being very concerned with COVID-19 saw increased odd of being risk adverse (OR = 3.07, 95%CI[1.93, 5.04], BH adjusted *p*-value = < 0.001) and having a household income of >$100,000 lowered the odds of being risk adverse (OR = 0.56, 95%CI[0.36, 0.87], BH adjusted *p*-value = 0.048).

**Table 2:** Model fit statistics (risk averse)

Model step	AIC	Pseudo *R*^2^
Individual factors	2209.3	0.08
Social factors	2067.4	0.15
Full model	1762.1	0.18

Note. Individual factors include age, gender, income, education, years lived in Canada, disability status, and phone ownership. Social factors include household composition, social cohesion, traffic safety, crime safety, and stranger danger. Full model includes the environmental factors, province of residence and ALE index scores

**Table 3:** Full model summary (odds of being risk averse)

Characteristic	OR^1^	95% CI^1^	BH *p*-value
**Child age**	0.94	0.87, 1.02	0.294
**Child gender**			
Boy	—	—	
Girl	0.89	0.70, 1.14	0.553
**Child Disability**			
No	—	—	
Yes	1.56	1.06, 2.34	0.097
**Child phone Ownership**			
Yes	—	—	
No	1.06	0.80, 1.40	0.904
**Time in Canada**			
Born in Canada	—	—	
2 years or less	2.13	1.54, 2.94	0.436
3 to 5 years	1.60	0.86, 2.89	0.292
6 years or more	1.83	0.74, 4.44	0.341
**Parent age**	1.00	0.98, 1.02	0.951
**Parent gender**			
Man	—	—	
Woman	0.72	0.55, 0.93	0.051
**COVID-19 concern**			
Not concerned	—	—	
Somewhat concerned	1.83	1.17, 2.96	0.051
Very Concerned	3.07	1.93, 5.04	**< 0.001**
**Parent education level**			
High school or less	—	—	
College	0.83	0.57, 1.20	0.503
University or more	0.94	0.66, 1.34	0.904
Prefer not to say	0.89	0.37, 1.97	0.904
**Household income**			
<$39,999	—	—	
$40,000-$99,999	1.05	0.72, 1.56	0.904
>$100,000	0.56	0.36, 0.87	**0.048**
Prefer not to say	0.53	0.27, 1.01	0.158
**Number of children at home**	0.80	0.69, 0.92	**0.001**
**Social cohesion**	0.87	0.72, 1.04	0.292
**Traffic safety**	0.98	0.78, 1.23	0.945
**Crime safety**	0.98	0.82, 1.16	0.904
**Stranger danger**	2.33	1.93, 2.82	**< 0.001**
**Region**			
BC/Yukon	—	—	
Prairies	0.98	0.63, 1.53	0.951
Ontario	0.84	0.57, 1.25	0.559
Quebec	1.62	1.06, 2.49	0.091
Atlantic Canada	0.68	0.33, 1.31	0.465
**Walkability score**			
1	—	—	
2	1.21	0.85, 1.72	0.492
3	1.42	0.99, 2.06	0.159
4	1.42	0.89, 2.25	0.294
5	0.78	0.41, 1.45	0.606

^1^ OR = Odds Ratio, CI = Confidence Interval, BH = Benjamini Hochberg

## Discussion

The aim of this study was to identify and examine correlates of risk aversion among a large sample of Canadian parents. Five factors were found to be significantly associated with risk aversion in our sample. Through a socio-ecological model lens, three of these were at the individual level. First, having a child born outside of Canada increased the odds of risk aversion when compared to having a child born in Canada. Risk perception is culturally dependent and may vary based on differences in adversities (e.g., structural racism and discrimination), cultural practices and values, and sociocultural stressors experienced by different racial/ethnic minority groups and generations [34]. For example, it was found that permissiveness among parents increased across immigrant generations [35]. Our findings align with recent work that found that language spoken at home was significantly associated with children’s outdoor time [36]. Contrary to previous studies that have found individual variables such as child and parental age and gender to determine parents’ willingness to let children engage in risky behaviours [21, 37–39], these factors were not found to be significant in this study.

Second, and independent of education level, parents with a household income greater than $100,000 had lower odds of being risk averse. We are not aware of any research that has examined the relationship between income/SES and parental risk tolerance. It is possible this finding reflects greater perceived safety of living in higher SES neighbourhoods or perhaps the financial flexibility to constantly supervise their children in ways that perceived risk is attenuated. Finally, greater concern about COVID-19 was associated with increased odds of risk aversion in our study. Qualitative studies have highlighted parental concerns about their children being able to adhere to public health protocols in the first wave of the COVID-19 pandemic [40]. This may influence parental perceptions of outdoor active play when other children are involved. It may also be that some parents have a disposition in having heightened concerns about their child’s safety that is generalizable across contexts or situations.

From the social environment, two factors were associated with parental risk aversion. Household composition was negatively associated with tolerance of risk, in that more children in the home was found to lessen the odds of risk aversion in parents. In the context of IM which might be reduced by parental concerns about risk, other studies have similarly demonstrated that children who can be accompanied by their older siblings, have greater IM [41–43]. Literature has shown that parents tend to demonstrate higher levels of control, discipline, and restrictions among first born children when compared to later born children, indicating the potential for greater risk aversion among households comprised of only one child [44]. Further, siblings tend to care and look out for one another, with older siblings frequently taking on roles as teachers and protectors of younger siblings [38, 45, 46]. Thus, the presence of an older sibling could help lessen parents’ risk perception.

Perceptions of social cohesion, traffic and crime safety did not differentiate the most risk averse parents from the other tolerance categories. Similarly, the walkability of the neighbourhood, that may influence opportunities for traffic exposure and interactions with other people, was not significant. Independent of concerns about COVID, the most critical factor associated with risk tolerance was concern about ‘stranger danger’. More broadly, ‘stranger danger’ is a common concern for parents regarding the IM of their children, particularly daughters [47–50]. That ‘stranger danger’ is so central to explaining risk tolerance in our sample is noteworthy. Heightened ideas of ‘stranger danger’ can arguably be traced back to the 1980s when an upsurge of widespread and sensationalized media coverage of missing children caused a mass panic among parents, as many believed that the incidence of abducted children was on the rise [51]. This “moral panic” has continued into present day parental fears [51]. In reality, child abductions are quite rare, with one report stating that only 1 case of child abduction by a total stranger was confirmed by the Royal Canadian Mounted Police over a two-year period in Canada [52]. This has been estimated as the likelihood of a child in Canada getting kidnapped being 1 in 14 million [53]. Further, crime statistics clearly show that it is not a stranger, but someone known to the victim, who is most likely to be their abductor [54].

While child abduction remains rare, statistics may not alleviate parental concerns. Wodda [51] draws on the work of Wayne Logan and his notion of ‘probability neglect’ which “encourages individuals to focus on emotionally charged negative occurrences, rather than their empirical likelihood” [55, pp. 406–407]. Emotional-based responses in interpreting strangers as potentially dangerous, rather than potentially good Samaritans, are a consequence. Such fears have contributed to increasingly risk averse parenting practices in the modern day, creating a “culture of fear disproportionate to the scope of the problem” [51] with the possibility that such fears may also be transmitted intergenerationally [56]. It may be crucial to work towards reframing these ideas of risk among parents so that children have opportunities for risky play and its developmental benefits [11].

Reframing parents’ ideas of risk to focus on the benefits to be gained from greater independence and engagement in outdoor active play may be most critical. In fact, reframing may be more salient than trying to reduce fears of child abduction and can be done through risk reframing interventions and educational campaigns. For example, the *OutsidePlay.ca* intervention tested a web-based intervention or an in-person workshop to reframe mothers’ perceptions of risk and change parenting behaviors [57]. The intervention was underpinned by social cognitive theory and asked participants to progress through a series of self-reflection exercises and development of a goal for change. For example, the first exercise (reflection) asked parents to consider the values and traits they most desired for their child in adulthood, their child’s favourite activities, their own favourite play activities at the same age, and what they got out of these childhood activities. Using the TriPS scale as the primary outcome measure, mothers in the web-based intervention had significantly higher tolerance of risky play at 1 week and 3 months after the intervention, and mothers in the in-person workshop had significantly higher tolerance of risky play at 1 week after the intervention, compared to mothers in the control condition [26]. While changes in behaviour were not assessed, these promising results demonstrate tolerance of risk is modifiable through reframing interventions.

The scalability of web-based interventions like OutsidePlay.ca is an attractive characteristic that lends itself to potential broader population reach. In tandem with social marketing campaigns promoting the benefits of outdoor play [58], there might be a start of a societal shift towards parents being more accepting and permissive of independent mobility and outdoor active play; however, this might be an optimistic expectation given the entrenched nature of the “stranger danger” myth in today’s society [51]. In line with other Canadian research exploring correlates of IM, a lower tolerance of risk and/or IM is observed among households where the child is not born in Canada or where English is not the primary language spoken at home [50]. Risk reframing interventions will need to consider cultural differences among target audiences to ensure their appeal, applicability, and impact [26].

### Strengths and limitations

There are limitations to this study that should be acknowledged. First, the cross-sectional design precludes conclusions about causality and the direction of associations between tolerance of risk and outdoor play for example. Second, there are likely unmeasured factors that may explain risk aversion. One factor may be traffic safety which was poorly measured in this study given the unsatisfactory alpha of the scale (0.53). It will also be important to further examine the influence of race and/or ethnicity and, by extension, social and cultural norms that may influence parent perceptions of risk. Third, the study took place in the first year of the COVID-19 pandemic and it is not clear how that context influenced participant reflections and responses to the survey. Lastly, our measure regarding COVID-19 concern does not convey any information about the nature of those concerns (e.g., their child interacting with other children; concerns about personal risk of exposure; concerns about health consequences). In contrast, strengths of the study include the large national sample, the inclusion of objective measures of walkability, and the assessment of correlates representing multiple levels of influence of the social-ecological model.

## Conclusion

Parental tolerance of risk is associated with several characteristics including income, concerns about COVID-19, number of children and time spent living in the country, but concerns about stranger danger remains the most notable safety-related factor. Tailored interventions that reframe perceptions of risk of outdoor active play for parents are needed. Such interventions could reframe concerns about stranger danger which persist despite occurrences of stranger abduction being extremely rare. A focus on the benefits of outdoor play and IM is warranted. Interventions could also be adapted for immigrant families and those with fewer children, as those that appear to struggle most with risk aversion. Additionally, longitudinal research should investigate changes in risk tolerance among parents to identify modifiable predictors of change over time.

## Electronic supplementary material

Below is the link to the electronic supplementary material.

Supplementary Material 1

## Data Availability

No datasets were generated or analysed during the current study.
