# Supplementary material for: Stranger danger or good Samaritan? A cross-sectional study examining correlates of tolerance of risk in outdoor play among Canadian parents
Source: BMC Public Health. 2025 Feb 14;25:627. doi: 10.1186/s12889-025-21848-8 (PMC11829390; doi:10.1186/s12889-025-21848-8)
Supplement: Supplementary file 1 — Supplementary Material 1 [file 12889_2025_21848_MOESM1_ESM.docx]

**Supplementary File: Parent questionnaire**

- This questionnaire should take about 20 minutes to complete.
- Please answer the questions as per the situation today to the best of your knowledge.
- Your answers will be made anonymous and kept confidential.

---------------------------------------------------------------------------------------------------------------------

1. Has anyone in your household been diagnosed with COVID-19 within the last two weeks?

□ No

□ Yes

□ No, but we are awaiting test results

1. Was your household under a self-isolation or quarantine order in the last week (i.e., not allowed to leave the house, due to travel or other potential exposure to COVID-19)?

□ No

□ Yes

□ We are self-isolating without being ordered

1. In which province or territory do you live?

□ British Columbia

□ Alberta

□ Saskatchewan

□ Manitoba

□ Ontario

□ Quebec

□ New Brunswick

□ Nova Scotia

□ Prince Edward Island

□ Newfoundland

□ Northwest Territories

□ Yukon

□ Nunavut

**The following questions ask about your child’s movement behaviours in the last week. If you have more than one child who is aged 7-12, please think about the child whose name is first alphabetically**.

1. What is your child’s first name? _________________________
2. Was [insert child’s name] school open in the last week?

□ Yes

□ No – go to question 6

1. How did [insert child’s name] attend school last week?

□ In person

□ Online

□ Blended (a mixture of in person at school and online)

1. Were school buses for [insert child’s name] school running in the last week?

□ Yes

□ No

□ Not applicable

1. Was [insert child’s name] affected by any of the following conditions in the last week? Please select all responses that apply.

□ Flu or cold

□ Asthma

□ Injury

□ Other. Please specify: ___________________________________________

1. How would you describe your current level of concern regarding COVID-19?

□ Not concerned

□ Somewhat concerned

□ Very concerned

1. How old is [insert child’s name] ? ___________ years
2. What gender does [insert child’s name] identify as?

□ Boy

□ Girl

□ They identify as [SPECIFY]

□ I’d rather not say

1. Does [insert child’s name] have a diagnosed disability or chronic condition?

□ Yes [specify]

□ No

1. In which country was [insert child’s name] born? ___________________________________
2. How many years has [insert child’s name] lived in Canada?

□ They were born in Canada

□ 2 years or less

□ 3 to 5 years

□ 6 years or more

1. What is the 6-digit postal code of [insert child’s name] primary residence? _______________

□ Prefer not to respond

1. What type of home is [insert child’s name] primary residence?

□ Low rise Apartment/Condo

□ High rise Apartment/Condo

□ Townhouse

□ Semi-detached house

□ Detached house

□ Something else [SPECIFY]

1. Please specify the number of adults and children in your household.

______ adults (18 years or older)

______ children (less than 18 years)

1. Does your family have a dog or dogs?

□ Yes

□ No

1. On average, how many total hours and minutes per day did your child watch TV, use the computer, social media and play inactive video games during their free time over the last week?

Weekdays (per day) _______ Hours (Cap at 23) AND _______ Minutes (Cap at 59) – Don’t know

Weekend (per day) _______ Hours (Cap at 23) AND _______ Minutes (Cap at 59) – Don’t know

**The next questions relate to physical activity…**

Moderate-to-vigorous physical activity is any activity that increases your heart rate and makes you get out of breath some of the time. Examples include sports, swimming, soccer, hockey, running, or cycling. Light physical activity is physical activity that does not result in sweat production or shortness of breath. Examples include mild stretching, playing with animals, and leisurely walking.

1. In the last week, how many hours and minutes did your child usually spend in moderate-to-vigorous physical activity in a 24-hour period?

Weekdays (per day) _______ Hours (Cap at 23) AND _______ Minutes (Cap at 59) – Don’t know

Weekend (per day) _______ Hours (Cap at 23) AND _______ Minutes (Cap at 59) – Don’t know

1. In the last week, how many hours and minutes did your child usually spend in light physical activity in a 24-hour period?

Weekdays (per day) _______ Hours (Cap at 23) AND _______ Minutes (Cap at 59) – Don’t know

Weekend (per day) _______ Hours (Cap at 23) AND _______ Minutes (Cap at 59) – Don’t know

1. In the last week, how many hours and minutes did your child usually spend sleeping in a 24-hour period (including naps but excluding time spent resting while awake)?

Weekdays (per day) _______ Hours (Cap at 23) AND _______ Minutes (Cap at 59) – Don’t know

Weekend (per day) _______ Hours (Cap at 23) AND _______ Minutes (Cap at 59) – Don’t know

1. On a typical **weekday** during the past week, how much time did your child spend playing outdoors (choose only one answer):

□ None at all

□ Less than 1 hour

□ 1 to less than 2 hours

□ 2 to less than 3 hours

□ 3 to less than 4 hours

□ 4 to less than 5 hours

□ 5 hours or more

1. On a typical **weekend day** during the past week, how much time did your child spend playing outdoors (choose only one answer):

□ None at all

□ Less than 1 hour

□ 1 to less than 2 hours

□ 2 to less than 3 hours

□ 3 to less than 4 hours

□ 4 to less than 5 hours

□ 5 hours or more

1. On a typical day, the MAIN part of your child’s journey TO school is made by…

□ Walking

□ Bicycle

□ Bus, train, streetcar, subway, or boat/ferry

□ Car, motorcycle, or moped

□ Other. Please specify: _________________________________________

□ Not applicable; my child attends school at home

1. On a typical day, the MAIN part of your child’s journey FROM school to home is made by…

□ Walking

□ Bicycle

□ Bus, train, streetcar, subway, or boat/ferry

□ Car, motorcycle, or moped

□ Other. Please specify: _________________________________________

□ Not applicable; my child attends at home

1. Is your child allowed to walk or bike home from school on their own when schools are open?

□ Yes

□ No

1. When going to places other than school that are *within walking distance*, is your child taken there or allowed to go on their own?

□ Usually goes on their own

□ Usually taken

□ Varies

1. Is your child allowed to cross main roads on their own?

***Please note****: This question is included for all parents. Please answer even if the answer seems obvious.*

□ YES

□ NO

1. Is your child usually allowed to go out *on their own* after dark?

□ YES

□ NO

1. a) Does your child have a bicycle?

□ YES

□ NO – go to question 35

b) Is your child allowed to cycle on main roads on their own?

□ YES

□ NO

1. Is your child usually allowed to travel on local buses on their own (other than a school bus)?

□ YES

□ NO

□ Not Applicable – There are no local buses in our neighbourhood

1. How far from home is your child allowed to roam on their own? *(Only tick one box)*

□ They are not allowed out on their own

□ Less than a 5 minute walk away from home

□ Within a 5-15 minute walk from home

□ More than a 15 minute walk from home

1. How far from home is your child allowed to roam on their own with friends and/or siblings (unaccompanied by an adult)? *(Only tick one box)*

□ They are not allowed out on their own

□ Less than a 5 minute walk away from home

□ Within a 5-15 minute walk from home

□ More than a 15 minute walk from home

1. Does your child have a mobile phone?

□ YES

□ NO

1. Please indicate how much you agree or disagree with each of the following statements.

| Statement | Strongly disagree | Somewhat disagree | Neutral | Somewhat agree | Strongly agree |
| --- | --- | --- | --- | --- | --- |
| 1. I am capable of supporting my child’s physical activity over the next week |  |  |  |  |  |
| 1. I will have an opportunity to support my child’s physical activity over the next week |  |  |  |  |  |
| 1. I am capable of restricting my child’s screen time to no more than 2 hours per day over the next week |  |  |  |  |  |
| 1. I will have an opportunity to restrict my child’s screen time to no more than 2 hours per day over the next week |  |  |  |  |  |
| 1. I am capable of supporting my child’s sleep over the next week |  |  |  |  |  |
| 1. I will have an opportunity to support my child’s sleep over the next week |  |  |  |  |  |

1. Do you agree or disagree with the following statements about your neighbourhood?

| Statement | Strongly disagree | Somewhat disagree | Neutral | Somewhat agree | Strongly agree |
| --- | --- | --- | --- | --- | --- |
| 1. People around my neighbourhood are willing to help their neighbours. |  |  |  |  |  |
| 1. This is a close-knit neighbourhood. |  |  |  |  |  |
| 1. People in my neighbourhood can be trusted. |  |  |  |  |  |
| 1. People in my neighbourhood generally don't get along with each other. |  |  |  |  |  |
| 1. People in my neighbourhood do not share the same values, attitudes or beliefs. |  |  |  |  |  |

1. Please select the answer that best applies to your neighbourhood.

| Statement | Strongly disagree | Somewhat disagree | Somewhat agree | Strongly agree |
| --- | --- | --- | --- | --- |
| 1. There is so much traffic along nearby streets that it makes it difficult or unpleasant for my child to walk (alone or with someone) in our neighbourhood. |  |  |  |  |
| 1. The speed of traffic on most nearby streets is usually slow (50 km/h or less). |  |  |  |  |
| 1. Most drivers go faster than the posted speed limits in our neighbourhood. |  |  |  |  |
| 1. There is a high crime rate in our neighbourhood. |  |  |  |  |
| 1. The crime rate in our neighbourhood makes it unsafe for my child to go on walks (alone or with someone) at night. |  |  |  |  |
| 1. I am worried about letting my child play outside alone around my home (e.g., yard, driveway, apartment common area) because I am afraid of them being taken or hurt by a stranger. |  |  |  |  |
| 1. I am worried about letting my child be outside with a friend around my home because I am afraid my child will be taken or hurt by a stranger. |  |  |  |  |
| 1. I am worried about letting my child play or walk alone or with friends in my neighbourhood and local streets because I am afraid my child will be taken or hurt by a stranger. |  |  |  |  |
| 1. I am worried about letting my child be alone or with friends in a local or nearby park because I am afraid my child will be taken or hurt by a stranger. |  |  |  |  |

1. Would you allow your child to do the following activities?

| Questions | Yes | No |
| --- | --- | --- |
| 1. Would you let your child jump down from a height of 3-4 metres? |  |  |
| 1. Would you allow your child to play chase (or tag) with other children? |  |  |
| 1. Would you trust your child to play by themselves without constant supervision? |  |  |
| 1. Would you let your child go head first down a slippery slide? |  |  |
| 1. Would you allow your child to continue playing if they get a few scrapes during play? |  |  |
| 1. Would you let your child have lots of challenges when they play at home? |  |  |
| 1. Would you let your child use a hammer and nail unsupervised? |  |  |
| 1. Would you let your child climb up a tree within your reach? |  |  |
| 1. Would you let your child walk barefoot across a floor after broken glass had been swept up? |  |  |
| 1. Would you let your child walk on slippery rocks close to water? |  |  |
| 1. Would you allow your child to play-fight other children with sticks? |  |  |
| 1. Would you encourage your child to try new things that involve some risk? |  |  |
| 1. Would you allow your child to engage in “rough and tumble” play? |  |  |
| 1. Would you let your child play near the edge of steep cliffs? |  |  |
| 1. Would you allow your child to play in the bush or forest out of your sight? |  |  |
| 1. Would you let your child experience minor mishaps if what they are doing is lots of fun? |  |  |
| 1. Would you let your child run close to an open fire? |  |  |
| 1. Would you let your child swim in the ocean close to the shore while you were watching from the beach? |  |  |
| 1. Would you allow your child to continue playing if there is the potential they may break a bone? |  |  |
| 1. Would you let your child play in a backyard unsupervised? |  |  |
| 1. Would you allow your child to climb a rock wall that goes straight down to the water? |  |  |
| 1. Would you wait to see if your child could manage challenges on their own before getting involved? |  |  |
| 1. Would you let your child climb as high as they want to in trees? |  |  |
| 1. Would you allow your child to ride a bicycle down a steep hill at full speed? |  |  |
| 1. Would you trust your child to play safely? |  |  |
| z. Would you allow your child to use a sharp knife? |  |  |
| aa. Would you let your child play in a backyard supervised? |  |  |
| ab. Would you let your child balance on a fallen tree more than 2 metres above the ground? |  |  |
| ac. Would you encourage your child to take some risks if it means having fun during play? |  |  |
| ad. Would you allow your child to climb up a tree beyond your reach? |  |  |

**CHANGE IN MOVEMENT AND PLAY BEHAVIOURS**

The World Health Organization (WHO) declared COVID-19 as a pandemic on March 11, 2020. For the following questions, we would like you to consider how your child’s behaviours changed as a result of COVID-19 and related restrictions.

1. Compared to before the COVID-19 outbreak and related restrictions, my child walks, bikes or uses other active means of transportation (e.g., scooter, skateboard or rollerblades) to go from place to place…

□ A lot less

□ A little less

□ About the same

□ A little more

□ A lot more

1. Compared to before the COVID-19 outbreak and related restrictions, my child’s independent mobility (e.g., their amount of freedom to move around in our neighbourhood without adult supervision) is…

□ A lot less

□ A little less

□ About the same

□ A little more

□ A lot more

1. Using the scale below, please select the number that best describes **how much distress** your child has been experiencing over the past week, including today.

0 (No Distress) → 10 (Extreme Distress)

**The following questions are about you:**

1. How old are you? ______________ years

1. What gender are you?

Man □

Woman □

Other, specify: □ _____________________

Prefer not to answer □

1. What is your current employment status?

Working full time □

Working part time □

Self-employed / freelance work □

Student □

Homemaker □

Unemployed □

Retired □

I can’t work because of COVID-19 □

Prefer not to answer □

1. À quel niveau se situe la dernière année de scolarité que vous avez terminée?

□ Elementary (7 years or less)

□ High school, general or vocational (8 to 12 years)

□ College (pre-university, technical training, certificate, accreditation or advanced diploma (13-15 years))

□ University certificates and diplomas

□ University Bachelor (including classical studies)

□ University Master's degree

□ University Doctorate (PhD)

□ Prefer not to answer

1. What is the last year of education that your partner has completed (if applicable)?

□ Elementary (7 years or less)

□ High school, general or vocational (8 to 12 years)

□ College (pre-university, technical training, certificate, accreditation or advanced diploma (13-15 years))

□ University certificates and diplomas

□ University Bachelor (including classical studies)

□ University Master's degree

□ University Doctorate (PhD)

□ Prefer not to answer

1. Among the following categories, which one best reflects the total **income**, before taxes, of all the members of your household in the last year?

□ $19,999 or less

□ Between $20,000 and $39,999

□ Between $40,000 and $59,999

□ Between $60,000 and $79,999

□ Between $80,000 and $99,999

□ $100,000 or more

□ Prefer not to answer

1. Does your household own or lease an automobile?

□ No □ Yes, 1 automobile □ Yes, 2 or more automobiles

1. In the last week, how many hours and minutes did you usually spend sleeping in a 24-hour period (including naps but excluding time spent resting)?

Weekdays (per day) _______ Hours (Cap at 23) AND _______ Minutes (Cap at 59) – Don’t know

Weekend (per day) _______ Hours (Cap at 23) AND _______ Minutes (Cap at 59) – Don’t know

1. In the last week, how many hours and minutes did you usually spend sedentary (e.g., sitting or reclining) in a 24-hour period?

Weekdays (per day) _______ Hours (Cap at 23) AND _______ Minutes (Cap at 59) – Don’t know

Weekend (per day) _______ Hours (Cap at 23) AND _______ Minutes (Cap at 59) – Don’t know

1. In the last week, how many hours and minutes did you usually spend in front of screens during your leisure time in a 24-hour period (e.g., watching TV and videos, playing video games, browsing the web, texting, etc.)?

Weekdays (per day) _______ Hours (Cap at 23) AND _______ Minutes (Cap at 59) – Don’t know

Weekend (per day) _______ Hours (Cap at 23) AND _______ Minutes (Cap at 59) – Don’t know

1. In the last week, how many hours and minutes did you spend engaging in moderate-to-vigorous physical activity in a 24-hour period?

Weekdays (per day) _______ Hours (Cap at 23) AND _______ Minutes (Cap at 59) – Don’t know

Weekend (per day) _______ Hours (Cap at 23) AND _______ Minutes (Cap at 59) – Don’t know

1. On a typical day, the MAIN part of your journey TO work is made by…

□ Walking

□ Bicycle

□ Bus, train, streetcar, subway, or boat/ferry

□ Car, motorcycle, or moped

□ Other. Please specify: _________________________________________

□ Not applicable; I work from home or do not work

1. On a typical day, the MAIN part of your journey FROM work to home is made by…

□ Walking

□ Bicycle

□ Bus, train, streetcar, subway, or boat/ferry

□ Car, motorcycle, or moped

□ Other. Please specify: _________________________________________

□ Not applicable; I work from home or do not work

***Thank you very much for your help* ☺**
